# A Case Report of Large Bilateral Pulmonary Embolism in a Patient With Janus Kinase 2 (JAK2) Positive Mutation

**DOI:** 10.7759/cureus.25482

**Published:** 2022-05-30

**Authors:** Zahid Khan, George Besis

**Affiliations:** 1 Acute Medicine, Mid and South Essex NHS Foundation Trust, Southend-on-Sea, GBR; 2 Cardiology and General Medicine, Barking, Havering and Redbridge University Hospitals NHS Trust, London, GBR; 3 Cardiology, Royal Free Hospital, London, GBR

**Keywords:** recurrent pulmonary embolism, systemic anticoagulation, dysponoea, ekos catheter, sinus tachycardia, massive pulmonary embolism, jak 2 mutation

## Abstract

Venous thromboembolism may be the primary presentation in patients with polycythaemia vera (PV) and essential thrombocythemia. Most patients get diagnosed with polycythaemia vera after presenting with venous or arterial thromboembolism in the first place. Most patients tend to develop thrombosis just before or at the time of diagnosis, and this risk decreases over time. Patients aged >60 years with a history of previous thrombosis, elevated haematocrit, and leukocytosis are most at risk of thrombosis. We report a case of a 74-year-old patient presenting with shortness of breath for three days. A computerized tomography pulmonary angiogram showed bilateral pulmonary emboli with right heart strain. He underwent emergency EkoSonic™ endovascular system-directed thrombolysis (EKOS™, Boston Scientific, Marlborough, MA). The patient tested positive for the Janus kinase 2 gene mutation (JAK2), met two major and one minor criterion for PV, and was discharged home on oral anticoagulation. The Janus kinase 2 (JAK2V617F) mutation is quite common in patients with polycythaemia vera, thrombocythemia, and myelofibrosis, and these patients are at risk of both arterial and venous thrombosis, hence they require long-term follow-up.

## Introduction

Venous thromboembolism may be the primary presentation in patients with polycythaemia vera (PV) and essential thrombocythemia, and the incidence of venous thromboembolism increases with age [1]. The Janus kinase 2 (JAK2V617F) mutation is the main molecular marker of the Philadelphia-negative chronic myeloproliferative neoplasms (MPN), responsible for 95% of polycythaemia; 50% of thrombocythemia vera; and myelofibrosis cases [2]. Myeloproliferative neoplasms are uncommon disorders, and polycythaemia vera (PV) is the most common myeloproliferative neoplasm with an annual incidence rate of 1-2/100,000 people [3]. PV is characterised by the presence of a Janus kinase 2 (JAK2) mutation, erythrocytosis, and panmyelosis on bone marrow biopsy and is associated with an increased risk of thrombosis based on age and other risk factors. PV is commonly associated with venous thrombosis: however, few cases of PV associated with intracardiac thrombosis have been reported [4]. PV has also been reported to be associated with portal vein thrombosis, eventually resulting in portal hypertension and liver cirrhosis [5]. Bone marrow histology may be helpful in distinguishing PV from secondary erythrocytosis [5]. We present a case report of a 74-year-old patient who presented with shortness of breath (SOB) for three days and was diagnosed with bilateral pulmonary emboli with right heart strain. The patient was also found to be positive for the JAK2 mutation, and he underwent EkoSonic™ endovascular system (EKOS™, Boston Scientific, Marlborough, MA)-directed targeted thrombolysis and showed significant improvement of his symptoms within 48 hours.

## Case presentation

We present a case of a 74-year-old male patient who has presented with shortness of breath for the past three days. Past medical history (PMH) includes hypertension (HTN), resected basal cell carcinoma (BCC) on the nose and neck, and the patient had nasal reconstruction surgery. Regular medications include amlodipine 5 mg once daily (OD). The patient denied any cough and fever, and there was no recent travel history. He was triple vaccinated against COVID-19. On arrival, his vital signs showed the respiratory rate of 27, heart rate of 110 bpm, oxygen saturation (SpO_2_) of 94% on 15 litres, and blood pressure of 110/60 mmHg. Besides SOB, he also had chest pain that was pleuritic in nature and was made worse by breathing.

On clinical examination, the chest was clear, heart sounds were normal, and Homan's signs was negative. Lab tests showed elevated D-dimer, troponin T, haemoglobin, and N-terminal pro-brain natriuretic peptide (pro-BNP) but low erythropoietin (EPO) levels of 1.94 mUI/mL (normal range: 2.6-18.5 mU/mL) (Table 1).

**Table 1:** Lab results for the patient. JAK2: Janus kinase 2.

Investigation/test	Day 1	Day 2	Day 3	Normal value
Haemoglobin	182	177	174	135-170 g/L
White cell count	10.48	10.83	8.22	3.5-11 × 10^9^/L
Neutrophils	7.99	7.83	5.95	1.7-7.5 × 10^9^/L
Platelet	145	166	187	150-400 × 10^9^/L
Mean cell volume	94.1	95.0	96.8	79-98 fL
Sodium	136	134	138	135-145 mmol/L
Potassium	3.7	3.4	3.7	3.5-5.1 mmol/L
Urea	8.1	7.8	6.8	2.9-8.2 mmol/L
Creatinine	90	77	73	66-112 umol/L
C reactive protein	72	85	87	0-5 mg/L
Fibrinogen	3.9	4.5	3.9	1.6-3.8 g/L
Prothrombin time	12.1	12.4	12.9	9-12 s
Activated partial thromboplastin time	52.7	52.9	53.4	24.7-37 s
D-dimer	>80,000	>60,000	>25,000	0-400 ng/mL
Troponin T	130	85	54	0-14 ng/L
JAK2	Positive	-	-	-
International normalised ratio	1.2	1.6	1.9	0.9-1.12 ratio
N-terminal pro-brain natriuretic peptide	7,136 ng/L	3075	1456	<400 ng/L

Chest radiography was unremarkable and a computerized tomography scan of pulmonary angiogram (CTPA) showed bilateral large pulmonary embolism extending up to the main pulmonary artery, evidence of right ventricular strain, and a right ventricle to left ventricle size ratio (RV: LV ratio) of 1.5:1. A bedside echocardiogram showed a dilated right ventricle without any evidence of left ventricular thrombus as shown in Video 1. 

**Video 1:** Echocardiogram apical four-chamber view shows a dilated right ventricle and an increased right ventricle vs left ventricle ratio.

**Figure 1:**
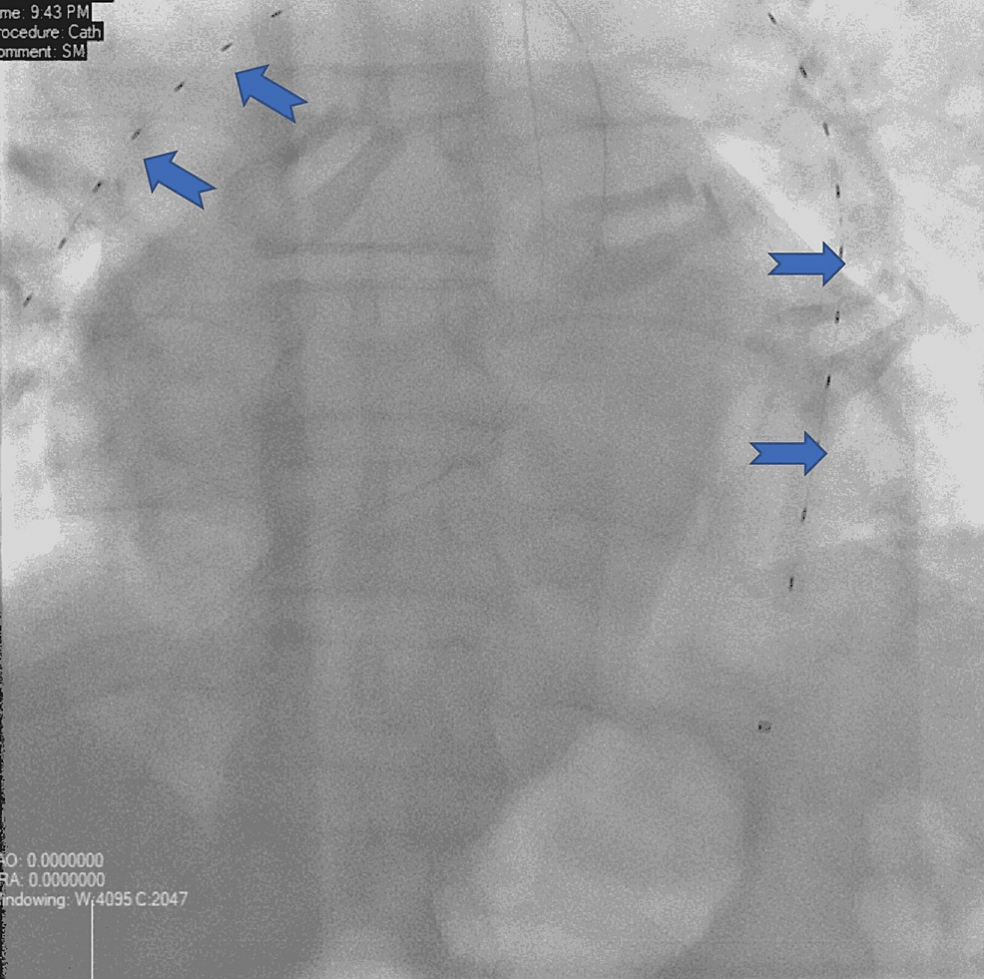
EkoSonic™ endovascular system-directed thrombolysis catheter system with ultrasound transducers used to direct thrombolytic agents at pulmonary embolism.

The patient received a treatment dose of unfractionated heparin. His pulmonary embolism severity index (PESI) score was 124, which puts him in class four high-risk category with a 30-day mortality of up to 11.4% based on the PESI score. The patient underwent emergency EkoSonic™ endovascular system-directed thrombolysis (EKOS) in view of a large pulmonary embolism with right ventricular strain and a high PESI score. The EKOS system is used to provide targeted local thrombolysis under ultrasound guidance as shown in Figure 1.

Patient's symptoms improved significantly within 24 hours, and the oxygen requirement was reduced significantly and was saturating at 95% without oxygen. He was commenced on apixaban 10 mg twice daily (BD) for a week, followed by 5 mg BD lifelong in view of pulmonary embolism secondary to PV. He was reviewed by a haematology consultant and advised to remain on lifelong anticoagulation. Clinical biomarkers showed improvement as shown in Table 1 and a repeat echocardiogram showed improvement in the size of RV. He was discharged home on day four with outpatient haematology follow-up.

## Discussion

PV is a chronic myeloproliferative disease characterised, and the diagnostic criteria used for its diagnosis, include World Health Organisation (WHO) guidelines or British Society of Haematology (BCSH) guidelines. According to WHO criteria for men, all three major criteria (Hb >16.5 g/dL or haematocrit >49% or increased red cell mass, bone marrow biopsy showing panmyelosis, and presence of JAK2V617F or JAK2 exon 12 mutations) or the first two major criteria and the minor criterion of subnormal serum erythropoietin (EPO) are necessary for the diagnosis to be made as shown in Table 2 [6]. The diagnostic criteria for men are slightly different than for women based on the haemoglobin and haematocrit values; otherwise, diagnosis requires the presence of both major criteria and one minor criterion or the presence of one major criterion together with two minor criteria in both genders.

**Table 2:** WHO diagnostic criteria for diagnosis of PV. WHO: World Health Organisation, PV: polycythaemia vera, JK2: Janus kinase 2. Source [7].

Category	2016 WHO diagnostic criteria
Major criteria	Haemoglobin >16.5 g/dL in men, 16.0 g/dL in women or haematocrit 49% in men, 48% in women or increased red cell mass. Bone marrow biopsy showing hypercellularity for age with trilineage growth (panmyelosis), including prominent erythroid, granulocyte, and megakaryocyte proliferation with pleomorphic, mature megakaryocytes, presence of JAK2 V617F, JAK2 exon 12 mutation, or other functionally similar mutations such as JAK2 exon 12 mutation
Minor criteria	Serum erythropoietin levels are below the reference range for normal. Diagnosis requires the presence of all three major criteria, or the first two major criteria plus one minor criterion. Haemoglobin or haematocrit greater than the 99th percentile of the method-specific reference range for age, sex, altitude of residence, or haemoglobin greater than 17 g/dL in men, and 15 g/dL in women, if associated with a documented and sustained increase of at least 2 g/dL from an individual's baseline value that cannot be attributed to correction of iron deficiency, or elevated red cell mass greater than 25% above mean normal predicted value.

The JAK2V617F mutation is present in the JH2-pseudokinase domain and exon-12 mutations such as K539L are mostly present in the region linking JH2 and JH3. It is important to remember, however, that about 17% of patients may have normal EPO levels initially and, hence, may not fulfil the diagnostic criteria. However, the EPO levels drop on repeat testing, thus fulfilling the diagnostic criteria [6]. This patient met two major criteria and one minor criterion as the patient had haemoglobin >180 g/dL, a JAK2 mutation, and a subnormal erythropoietin level.

Patients with MPNs have an increased risk of thrombotic events, and these events increase the risk of mortality and morbidity in these patients. Both thromboembolism (TEs) and cardiovascular complications are more prevalent in PV compared to other myeloproliferative polycythaemia vera (MPV) disorders [8]. A retrospective study based on a total of 3001 PV patients reported that PV patients have a three- and 13-fold higher risk of arterial thrombosis and venous thrombosis, respectively, compared with controls matched for age and gender at three months after initial diagnosis [9]. Thrombosis and thromboembolism (TEs) are observed in approximately 39-41% of patients with PV, and arterial thrombosis comprises 60-70% of all cardiovascular events in patients with PV. Arterial thrombosis in these patients may present as a transient ischaemic attack (TIA), cerebrovascular accident (CVA), acute myocardial infarction (AMI), and peripheral arterial occlusion [10]. The Gruppo Italiano Studio Policitemia study based on 1213 patients who were followed up for 20 years reported that approximately 64% of thromboembolic events occurred shortly before or at the time of diagnosis, and the reported incidence is about 12-15% just before the diagnosis [11]. Similar findings were reported by the European Collaboration on Low-Dose Aspirin in Polycythaemia Vera (ECLAP) study, in which two-thirds of all events occurred shortly before or at the time of diagnosis [12]. There are a few published case reports of elevated EPO levels associated with PV and Budd-Chiari syndrome (BCS) [4]. In another case report [13], the JAK2 mutation was reported to be associated with thromboembolism in a young patient without the myeloproliferative disorder. JAK2 mutation sometimes can be falsely negative initially and in a case report of a 40-year-old patient with a positive family history of deep venous thrombosis (DVT), she developed thrombosis of the inferior vena cava extending to the suprahepatic veins and pulmonary arteries, although she tested negative for JAK 2 mutation initially but tested positive later [2]. PV can also be associated with intracardiac thrombus and a case report of a 60-year-old patient was found to have left apical thrombus after presenting with tinnitus and vertigo [3]. Several factors increase the risk of thrombosis in patients with PV, including increased haematocrit, impaired fibrinolytic activity, platelet activation, leukocyte activation, endothelial damage, and increased whole-blood viscosity [3]. Key risk factors for intracardiac thrombus in these patients include valve disease, prosthetic valve, and cardiomyopathy [3,14]. Studies have shown that the key risk factors associated with recurrent thrombosis include old age, the presence of cardiovascular risk factors such as hypertension, diabetes mellitus, hypercholesterolaemia, atrial fibrillation, smoking, and previous thromboembolic events. A study of 494 patients reported that only age >60 years and previous thromboembolic events were associated with a higher recurrence rate [15,16].

## Conclusions

In conclusion, the Janus kinase 2 mutation polycythaemia vera is frequently associated with venous thrombosis and has also been reported to cause intracardiac and portal vein thrombosis. EkoSonic™ endovascular system-directed thrombolysis is effective in short-term symptomatic relief of patients with large pulmonary embolism, but long-term benefits need further studies. These patients should be commenced on life-long anticoagulation and require regular specialist follow-up.

## References

[1] Za T, Fiorini A, Rossi E, Ciminello A, Chiusolo P, Leone G, De Stefano V (2009). Prevalence of the JAK2 V617F mutation in patients with unprovoked venous thromboembolism of common sites and without overt myeloproliferative neoplasms. Br J Haematol.

[2] Salort A, Seinturier C, Molina L, Lévèque P, Imbert B, Pernod G (2014). Recurrent deep vein thrombosis and myeloproliferative syndrom: emergence of JAK2 mutation five years after the initial event. J Mal Vasc.

[3] Gangadharamurthy D, Shih H (2013). Intracardiac thrombosis in polycythemia vera. BMJ Case Rep.

[4] Gameiro RS, Rodrigues A, Gonçalves FM, Graça JP (2017). Bumpy road to the diagnosis of polycythaemia vera. BMJ Case Rep.

[5] Karakulska-Prystupiuk E, Gierej B, Paszkowska-Kowalewska M, Wilkowojska U, Jedrzejczak WW (2012). Portal vein thrombosis as the main symptom of unclassified JAK2-positive myeloproliferative neoplasm--case report. Pol Merkur Lekarski.

[6] Arber DA, Orazi A, Hasserjian R (2016). The 2016 revision to the World Health Organization classification of myeloid neoplasms and acute leukemia. Blood.

[7] (2022). Polycythemia vera diagnostic criteria. https://www.wikidoc.org/index.php/Polycythemia_vera_diagnostic_criteria.

[8] Barbui T, Finazzi G, Falanga A (2013). Myeloproliferative neoplasms and thrombosis. Blood.

[9] Vannucchi AM (2010). Insights into the pathogenesis and management of thrombosis in polycythemia vera and essential thrombocythemia. Intern Emerg Med.

[10] Tefferi A, Elliott M (2007). Thrombosis in myeloproliferative disorders: prevalence, prognostic factors, and the role of leukocytes and JAK2V617F. Semin Thromb Hemost.

[11] Gruppo Italiano Studio Policitemia (1995). Polycythemia vera: the natural history of 1213 patients followed for 20 years. Ann Intern Med.

[12] McMullin MF, Harrison CN, Ali S (2019). A guideline for the diagnosis and management of polycythaemia vera. A British Society for Haematology Guideline. Br J Haematol.

[13] Kim JJ, Kwon SS, Lee HJ, Lee HY, Jeong MH, Kim YH (2009). A case of pulmonary thromboembolism with JAK2 mutation. Tuber Respir Dis.

[14] Pearson TC (2002). The risk of thrombosis in essential thrombocythemia and polycythemia vera. Semin Oncol.

[15] De Stefano V, Za T, Rossi E (2008). Recurrent thrombosis in patients with polycythemia vera and essential thrombocythemia: incidence, risk factors, and effect of treatments. Haematologica.

[16] Marchioli R, Finazzi G, Landolfi R (2005). Vascular and neoplastic risk in a large cohort of patients with polycythemia vera. J Clin Oncol.

